# Immune Tolerance vs. Immune Resistance: The Interaction Between Host and Pathogens in Infectious Diseases

**DOI:** 10.3389/fvets.2022.827407

**Published:** 2022-03-29

**Authors:** Hafiz Ishfaq Ahmad, Abdul Jabbar, Nadia Mushtaq, Zainab Javed, Muhammad Umar Hayyat, Javaria Bashir, Iqra Naseeb, Zain Ul Abideen, Nisar Ahmad, Jinping Chen

**Affiliations:** ^1^Department of Animal Breeding and Genetics, University of Veterinary and Animal Sciences, Lahore, Pakistan; ^2^Department of Clinical Medicine, Faculty of Veterinary Science, University of Veterinary and Animal Sciences, Lahore, Pakistan; ^3^Department of Biological Sciences, Faculty of Fisheries and Wildlife, University of Veterinary and Animal Sciences, Lahore, Pakistan; ^4^Institute of Pharmaceutical Sciences, Faculty of Biosciences, University of Veterinary and Animal Sciences, Lahore, Pakistan; ^5^Department of Medical Sciences, Sharif Medical and Dental Hospital, Lahore, Pakistan; ^6^Institute of Microbiology, Faculty of Veterinary Science, University of Veterinary and Animal Sciences, Lahore, Pakistan; ^7^Department of Zoology, Ghazi University, Dera Ghazi Khan, Pakistan; ^8^Department of Livestock Management, University of Veterinary and Animal Sciences, Pattoki, Pakistan; ^9^Guangdong Key Laboratory of Animal Conservation and Resource Utilization, Guangdong Public Laboratory of Wild Animal Conservation and Utilization, Institute of Zoology, Guangdong Academy of Sciences, Guangzhou, China

**Keywords:** disease resistance, immune tolerance, pathogens, adaptive immunity, infectious diseases

## Abstract

The immune system is most likely developed to reduce the harmful impact of infections on the host homeostasis. This defense approach is based on the coordinated activity of innate and adaptive immune system components, which detect and target infections for containment, killing, or expulsion by the body's defense mechanisms. These immunological processes are responsible for decreasing the pathogen burden of an infected host to maintain homeostasis that is considered to be infection resistance. Immune-driven resistance to infection is connected with a second, and probably more important, defensive mechanism: it helps to minimize the amount of dysfunction imposed on host parenchymal tissues during infection without having a direct adverse effect on pathogens. Disease tolerance is a defensive approach that relies on tissue damage control systems to prevent infections from causing harm to the host. It also uncouples immune-driven resistance mechanisms from immunopathology and disease, allowing the body to fight infection more effectively. This review discussed the cellular and molecular processes that build disease tolerance to infection and the implications of innate immunity on those systems. In addition, we discuss how symbiotic relationships with microbes and their control by particular components of innate and adaptive immunity alter disease tolerance to infection.

## Introduction

The immune system has evolved, which increases the survival of humans against invading pathogens in the body. There were limitations in innate immunity, such as no-self and non-self-antigen and no memory function (1). These have been evolved into an acquired immunity which offers a high defense and protection against pathogens with memory functioning for preventing the host from future attack by some pathogens (2). A mechanism of immune tolerance defends the self-antigen from the immune system's destructive response, which usually performs against non-self-antigen (3). Immune tolerance is of two types, i.e., Central immune tolerance and Peripheral immune tolerance. These mechanisms involve the T regulatory cells from the thymus and periphery, respectively (4). Different immune organs called primary (thymus and bone marrow) and secondary (spleen and lymph nodes), depending on their function in the immune development, are crucial in the process. The immune defensive cells comprise macrophages, dendritic cells (DCs), natural killer (NK) cells, T and B cells, mainly involved in this mechanism. Various pathways lead to activation of the immune system other than tolerance and defense mechanisms. Immune checkpoints proteins and receptors are performing actions in activation of the immune response, “TLR, NLRs (nucleotide-binding oligomerization domain-like receptors), pattern recognition receptors (5), CLRs (C-type lectin receptors) (6), and AIM2 (absent in melanoma two protein)” (7) and many others are checkpoint receptor proteins. The immune cells are programmed during their fetal development, but they can be reprogrammed afterward during puberty (8). Different immune cell populations have come to view different periods of life by differentiation, and during differentiation, the chances of reprogramming (imprinting) are persistent (9). In the last years, long-term reprogramming of cells play a role in immune paralysis, disease tolerance, and cytokine production after the pathogenic attack (10). The immune system targeting to treat different immunological disorders has been revolutionized by using various checkpoint inhibitors as medicines (11). However, some challenges are faced in studying the immune tolerance and resistance, like the use of some drugs decreases the number of the immune cells, leading to the reduction in their functionality (12).

The cellular and molecular processes that create disease tolerance to infection and the consequences of innate immunity on those systems have been discussed in this article. We also go through how symbiotic connections with bacteria and their management by specific components of innate and adaptive immunity affect disease tolerance to infection. Innate and acquired immune systems involved in pathogenic killing and illness management caused by these pathogens will be heavily focused. The change and tuning of cellular programming are also thought to promise to cure immune-mediated illness. By elucidating the possible involvement of acquired immunity in disease pathophysiology, new steps toward understanding disease pathogenesis can be taken, perhaps leading to novel treatment approaches.

## Evolution of the Immune System

With the growth and development of a human child, the immune system is evolved with time, but there is still a danger of attack by pathogenic microorganisms (2). The ancestors of the successors provided a more sophisticated system. That can fight overall pathogens virtually, ensuring that the protection is for the long run and ensuring no self-attack on the human body. This system is called the immune defense system. The least useful mechanisms have been discarded and left the best is innate immunity, which has evolved now (13).

### Innate Immunity and Its Limitations

This defense system is inherited from generation to generation and passed from the evolutionary time with remarkable changes. The innate defense system includes the following components, TLRs, complement system, and phagocytic cells. These components are not enough to protect a body from invading pathogens (1). This immune system performs a quick action against pathogens but has no short-term memory to protect This system has further loopholes, i.e., TLR receptors do not have the molecular ability to make a difference between self and non-self-antigens and initiate a defense response against both of them (14). However, the innate cells destroy the tissues in the surroundings and the whole organism. For instance, a typical innate cell, i.e., neutrophils in an unlucky patient's renal abscess, grows without check and balance (1). In response to infections with microbial pathogens, the immune system evolved over millions of years, consisting of cells, the substances they make, and the organs that coordinate those components. Its vital function in preserving health is based on its ability to distinguish, eliminate or manage invading microorganisms (15). The immune system's ability to identify foreign and hazardous intruders from self-components is critical to its protective role. The immune system is engaged in the prevention of cancer by monitoring and detecting self-cells that express new antigens, as well as the resolution and repair of tissue damage, in addition to its contributions to host defenses (16). Briefly stated, innate immunity gives evolutionary knowledge on the biological correlates of structures with which its receptors interact, i.e., structures that are likely to be linked with infectious organisms. Its limitations include the fact that it can recognize just a small number of microbiological structures that are well preserved and that it cannot develop at the same rate as microorganisms (17).

### Development of Acquired Immunity

These pressure condition of innate immunity limitation leads to alternative phenotypes in the major histocompatibility complex. This complex presents antigens to T and Natural Killer cells (adaptive immunity) and mainly regulates the immune responses (2). In a short time, new T cells and B cells are created as lymphocytes (1). Over approx. Five hundred million years ago, the genetic makeup of these two cells was present invertebrates of both having jaws and not having jaws (18, 19). Antibodies Igs present on B cells and T-cell receptors are on T cells for antigen recognition are known as cell surface receptors. These can help distinguish between self and non-self-antigens (20). T cells further divide into Helper T cells and Effector T cells. The B cells produce antibodies formed by plasma cells. Each has secreted molecules (cytokines) (21). At birth, CD45RA glycoproteins are present in all the T cells, which the naive T cells do not have a never attack by foreign antigens (2). Helper T cells dictate a strategy of defense response against a pathogenic attack, and Cytotoxic T cells cause the death of those cells, which give shelter to pathogens. After antigens' attack, lymphocytes start prolific proliferating and increasing their fight power and divide into their subsets to produce a more intense response (1). The number of T regulatory cells starts decreasing, and the number of memory cells increases gradually, equal to naive T cells number. For developing T helper 17 cells, the segmented bacteria are necessary (2) and the species of Clostridium which induces the colonic T regulatory cells (22, 23). Some immunological deficiencies are corrected by inducing the normal gut flora to germ-free animals. These animal data show that the various normal flora develops both memory T and B cells. The Navy cells have specific antibody receptors for the antigen in length north and detect a partial signal (24). The antigen bound with those receptors is internalized, and lysosomes present digested this complex. Human leukocyte antigen (HLA) Class II molecules presented on the cell surface, T follicular cells with their receptors signal B cells (25). These signals divide the B cell to produce antibody somatic hypermutation. Mutated antibodies B cells are favored to bind with immunogens because they have a higher affinity for binding (26). T cells do not perform somatic hypermutation, but somehow the genes that produce antibody-like receptors of T cells are present, but they have no advantage (2). B1a and B-2 cells produce antibodies and target different antigens. The IgM antibodies, which are two types, germinal center dependent and T dependent, are produced by B2 cells. They also produced IgG antibodies with high affinity (27). In contrast, B1a produces so-called natural antibody responses. The constitutive IgG and IgM antibodies start producing during fetal life and can also be detected in serum. These antibodies produce a response to antigen and trees in the body, which may be T dependent and T independent to phosphorylcholine. It depends upon the antigen presented form (28).

### The Immune System of Old Aged People

Due to lymphopenia with age, the number of autoimmune diseases increases because the immune system gets sacrificed, and immune tolerance is decreased (29). The immune system of an aged organism is similar to the newborn organism because of the reduction in innate cells' antimicrobial capacity, Dendritic cells' reduced capacity to present antigen, Natural killing cells reduced killing power, and compromised adaptive immunity. The immune system evolution within individuals elaborates the role of adults for the potential of survival in the species (2). Pathogens can be effectively combated when a strong innate immune system. Excessive reactions might be harmful; thus, they must be strictly regulated. A condition known as “inflamm-aging” occurs when an organism's immune system becomes increasingly proinflammatory as it gets older. This is common in a wide range of animals (30). Chronic inflammation contributes to the onset and progression of age-related disorders such as osteoporosis, atherosclerosis, and neurodegeneration, now well-established knowledge (31). Reports of increased inflammatory activity in old age appear to contradict research indicating functional abnormalities within the innate immune system cells at first glance. Cellular functional deficiencies can build up over time and prevent the body from eliminating infections, leading to a persistent failure to activate certain immune responses (32). For example, polymorphisms in genes that code for cytokines like interleukin 6, interleukin 10, and interferon-gamma are connected with variations in longevity and may influence the intensity of inflammatory processes as people age (33).

Neutrophil granulocyte function is well known to vary with age. When exposed to GM-CSF and Gram-positive bacteria, superoxide anion generation decreases with age. Changes in membrane fluidity, chemotaxis to specific chemoattractants, and other signal transduction pathways have also been discovered. In old age, neutrophilic granulocytes' bactericidal activity is diminished due to these abnormalities (34).

## Immune Tolerance

The immune system is the defensive system within a living body that majorly differentiates between allogenic (self) and xenogeneic (non-self) harmful particles known as “antigens” (35). The immune system then activates various responses to remove antigens from the body. To activate the proper immune response, multiplication and specialization of immune cells initiate after recognizing foreign invaders (36). However, in the case of self-antigens (originated within the body), the immune system shows a specific type of response termed as “immune tolerance” that is low or no immune response but continues usual response against non-self-antigens. Immune tolerance is developed by inducing specific antigens called “tolerogens” (3).

### Immune Tolerance Mechanisms

The tolerance of the immune system is important in maintaining immune homeostasis. Immune tolerance is carried out through Central Tolerance and Peripheral Tolerance (37). The T-regulatory cells directed from the thymus (Treg), regulate the central tolerance. However, the T-regulatory cells directed peripherally regulate the peripheral tolerance after the central tolerance (4).

## Central Immune Tolerance

**Figure 1:**
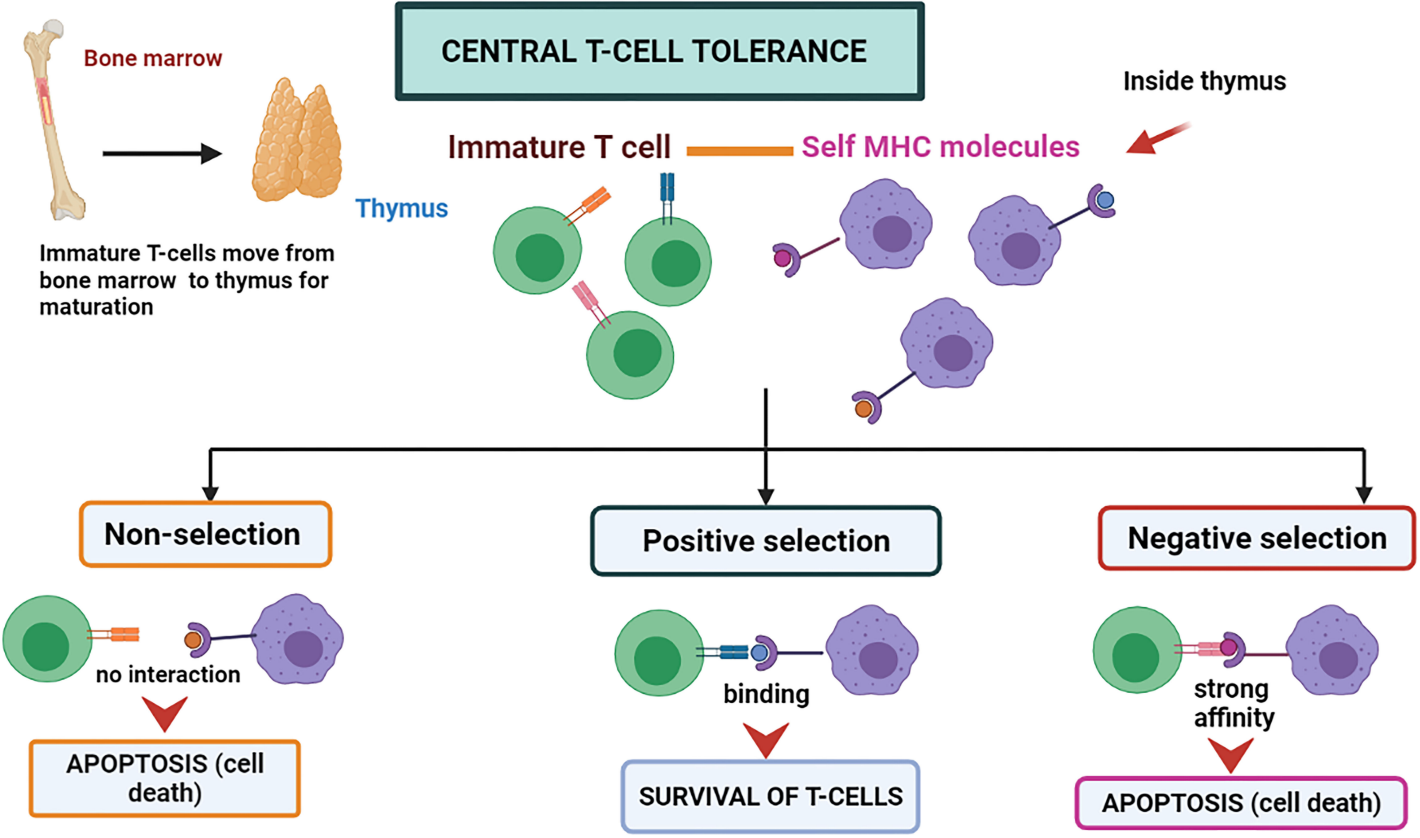
Central T-cell tolerance mechanism. The T-cells mature in the thymus by attaching themselves to self MHC molecules. If the interaction is not strong, the T-cell survives called “positive selection.” If the interaction is too strong, it leads to programmed cell death called “negative selection”.

Central immune tolerance is majorly performed in the thymus and bone marrow parts (organs and tissues of CIT) of the host body. During the development of an embryo and neonate stages, the T cells and immature B cells obtain tolerance against self-antigens by the selection processes, which may be positive or negative at the maturation time (38). During the process, the self-antigen receptors possessing cells are eliminated. T cells that do not recognize either major histocompatibility complex (MHC)-I and MHC-II molecules degenerate through apoptosis and are known as “positive selection” (38) (Figure 1). The high-affinity binding of T-cells to Major Histocompatibility complexes I, II, or I/II self-peptide complex are also directed to programmed cell death and are known as “negative selection” (38). A similar negative selection process has been observed during B cell development in the bone marrow. The B-cells possessing antibodies that are autoreactive on their surface are induced to programmed cell death. The process can be named clonal deletion (39). The immune system is assimilated to differentiate the self and non-self by deleting the autoreactive T and B-cells before they fully mature into functional immune cells by these central tolerance phenomena (3).

### Peripheral Immune Tolerance

**Figure 2:**
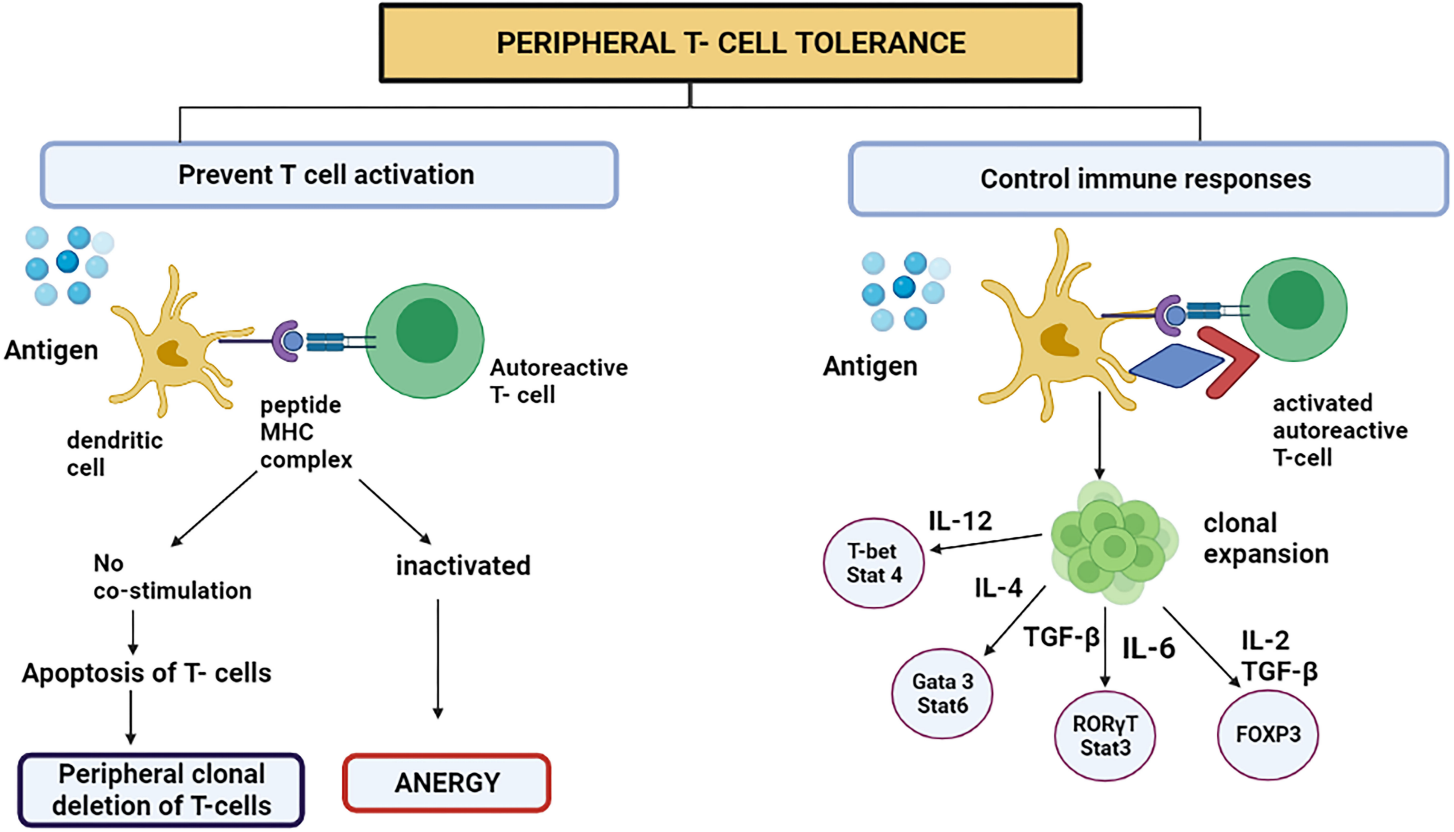
Peripheral T cell tolerance prevents T cell activation or controls the immune responses by switching on certain signaling pathways. However, to present T cell activation, the DC representing antigen either attaches to autoreactive T cell or starts apoptosis because the stimulatory component was absent on DC. This is called peripheral clonal deletion of T cells. If the T cell is inactivated, the process is called anergy.

This process mainly occurs in adults. This mechanism averts the immune system to overreact against environmental factors (40). The CD4^+^ Foxp3-type 1 Treg cells have received growing medical research attention. Tr1 cells are prompted by the chronic triggering of CD4^+^ T cells *via* antigen in the company of interleukin-10 (IL-10) (40). They signify a new subclass of CD4^+^ T cells in human beings plus mice. The immunomodulatory utilities of Tr1 cells style them as an encouraging objective for handling autoimmune diseases like cancer and avoiding organ transplant incompatibility (4). In 2013, the surface proteins which are characteristics for Tr1 cells in humans and mice were acknowledged (CD4^+^ CD49b^+^ LAG-3^+^ CD226^+^) (41). At the minimum, four important mechanisms have been known in the Tr1 cell function. First, T cells and antigen-presenting cells (APC) are suppressed by Tr1 cells, principally utilizing the secretion of IL-10. The immunomodulatory expression molecules such as immunoglobulin-like transcript-3 (ILT3), immunoglobulin-like transcript-4, and Human leukocyte antigen (HLA-G) on the dendritic cells are up-regulated by initiating the trail flanking the IL-10/IL-10R (42). The Tr1 cells are believed to be performing meaningful actions in treating and avoiding the immune diseases in which the system mistakenly attacks the body's cells, organ transplantation, and prolonged inflammatory diseases mainly by overturning the effector T cells and the memory cells response produced after the first attack and by regulating the peripheral immune tolerance (Figure 2) (4).

The basophils and mast cells are desensitized, which is among the initial effects after a successful AIT (allergen-specific immunotherapy). The induced Treg cells produce interleukin 10 (IL-10) and consequently transform the Transforming growth factor-beta (TGF-β) that, as a result, clamp down the effector cells that are involved in the inflammation due to allergens (Figure 2) (43).

Human Breg cells have been found among immature transitional B cells (identified as CD19^+^CD24^hi^CD38^hi^) (44). Human B_R_1 cells are categorized by a CD73^−^CD25^+^CD71^+^ phenotype and have been considered in the context of allergen tolerance induction (45). Breg cells might also perform a fundamental role in inducing tolerance toward allergens. Quite a few researches have revealed that B cells can quash allergen-mediated inflammation from the end-to-end secretion of IL-10 and TGF-β, thus suppressing effector T-cell reactions and inducing Treg cells. Additionally, Breg cells might endorse allergen tolerance with the privileged fabrication of IgG_4_ antibodies on differentiation en route for plasma cells (44). IgG_4_has various features that might propose a character in immune tolerance. Lacking the Fc receptor causes IgG_4_ not to function in antibody-dependent cellular cytotoxicity (46). Furthermore, IgG_4_ inflammation is restricted due to failure to fix a match and lessen allergy, consequently contending with IgE as a filibustering antibody for allergen obligatory to IgE Fc receptor-expressing cells (47, 48).

### Immune Defense

The immune defense system consists of immune cells, products, and humoral factors, i.e., complement proteins. The cellular products include antibodies, various growth factors, and cytokines. The humoral and immune cellular elements are responsible for fighting against the attack of harmful foreign microorganisms and removing them from the host body (49). The innate immune system has made evolutionary changes in the immune system. The innate immune system consists of various components. Skin acts as a barrier, small complement molecules, and different cells of innate immunity (50). The innate immune defense system protects the host body from foreign pathogens without the help of necessary conditions from the environment (51). The innate immune system quickly kills the pathogen and removes it from the host body upon the pathogen's entry into the body. Besides Metchnikoff's discovery of immunity by immune cells, many researchers have examined that body fluid also plays an important role in protection against pathogens causing diseases (52). In 1890, Antibodies were discovered by scientists Emil von Behring and Shibasaburo Kitasato. Humoral immunity comprises antibodies and cytokines and complement proteins (53). On the contrary, cellular mediated immunity comprises cellular fraction. Over time, the evolution in the immune defense system enables it to perform a cellular action against a pathogen known as adaptive immunity (54).

### Component of Immune Defense

#### Immune Organs

The origin of all blood cells is the stem cell of hemopoiesis present in bone marrow, but these blood cells mature and reside in different sites (55). Cells involved in the innate immune defense system are produced from bone marrow and reside in tissue and blood while the acquired immune defense cells T cells and B cells mature at different sites. T cells recombine their receptors in the thymus while B cells combine and mature in the bone marrow (56). The thymus and Bone marrow are believed to be the primary immune organs. After maturation of T and B cells in their respective primary immune organs, they move toward the lymphatic tissue sites where they reside (57). These cells in the lymphatic tissue sites are called lymphocytes. Lymphatic tissue sites of T and B cell residence called secondary immune organs. The various immune organs that are secondary include tonsils, lymph nodes, appendix, spleen, adenoids, Peyer's patches, and other MALT (mucosal-associated lymphoid tissue) (54).

#### Immune Cells

There are two categories of immune defense cells, i.e., the innate immune cells and adaptive immunity cells. Innate immune cells comprise granulocytes with granules (polymorphonuclear cells), mast cells, macrophages, and dendritic cells (58). Dendritic cells play a role in activating adaptive immunity cells, T and Treg cells, B cells, and NK cells (59). The innate immune response cells act immediately upon encounter with pathogens and have no immunological memory function. The adaptive immunity cells perform a delayed action which may take several days and develop an immunological memory (54).

## Mechanism of Immune Defense

**Figure 3:**
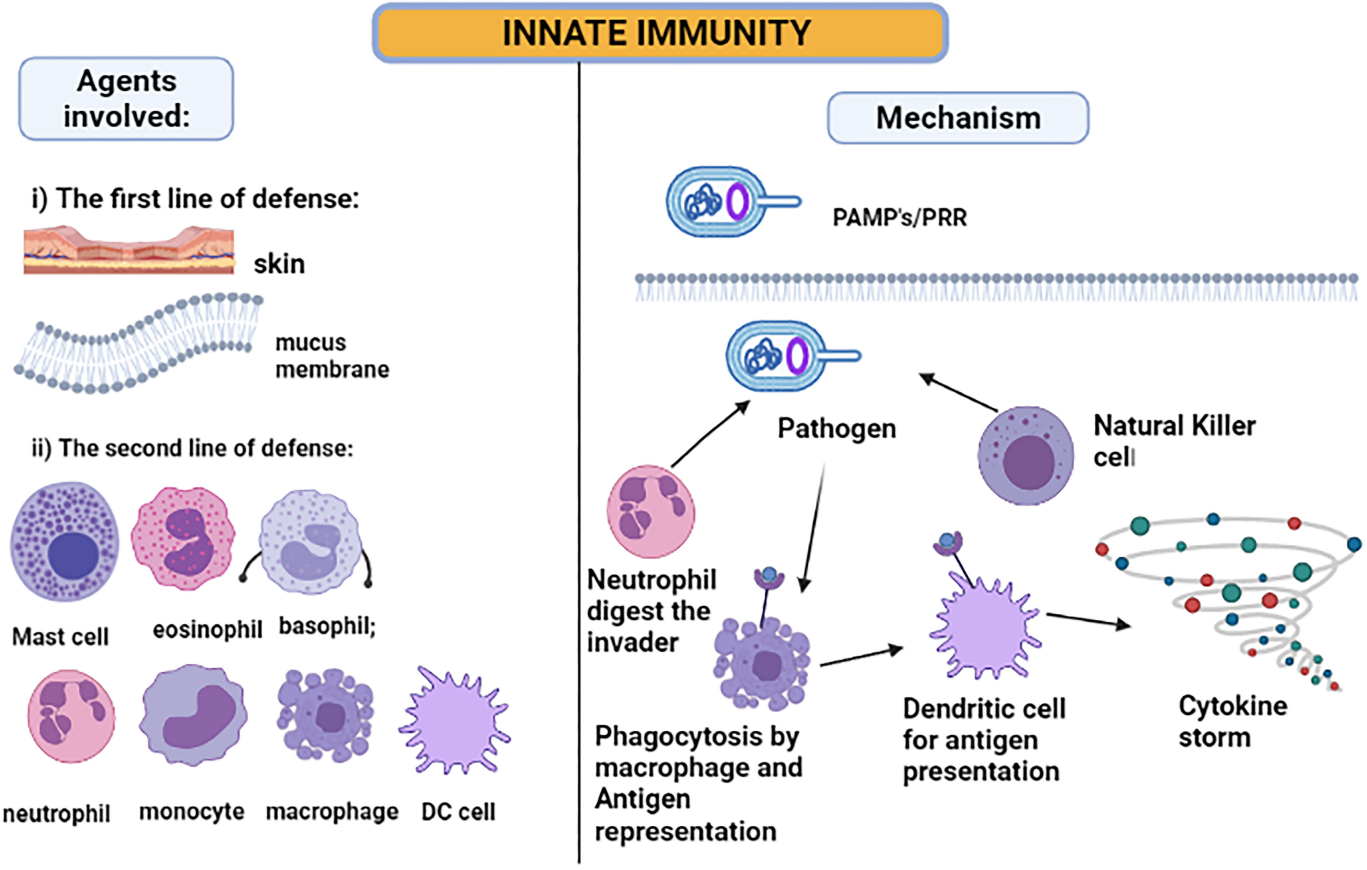
Innate immunity: pathogen-associated molecular patterns are recognized by Toll-like receptors. When the pathogen invades the host cell, the neutrophil and natural killer cells start digesting the pathogen. However, the macrophages and DC phagocytose the pathogen and act as antigen-presenting cells, producing a cytokine storm for attracting other immune cells to amplify the response.

The skin and the membranes surrounding cavities in the human body serve as the first line of defense when exposed to a disease-causing agent—a pathogen. The innate immune system is activated immediately after the infection (54), as illustrated in Figure 3. The inflammatory responses are modulated by the cytokines released due to local immune responses being affected by the keratinocytes and melanocytes of the skin cells (60). Hence, the skin serves as the major immunologically complex organ which possesses and ability to react or show response to any infectious or non-infectious foreign agent by modulating either the innate or the adaptive mechanisms of immunity (61, 62). For the intestine immune system, the epithelial goblet cells and enterocytes serve the main role in producing mucus and enhancing the immune response (63). Next, the macrophages, dendritic cells, and neutrophils will be activated when the pathogen-associated molecular patterns (PAMPs), lipopolysaccharide, flagellin, and ssRNA, bind to Toll-like receptors on the cell surface (59). These innate immune cells mainly show the response by phagocytosing the foreign particle when bound to the danger signal. The activated cells amplify the inflammatory response by releasing cytokines produced by activated macrophages and interferons (IF). The cytokines such as IFNα and IFNβ can develop resistance against viral pathogen infection (54).

**Figure 4:**
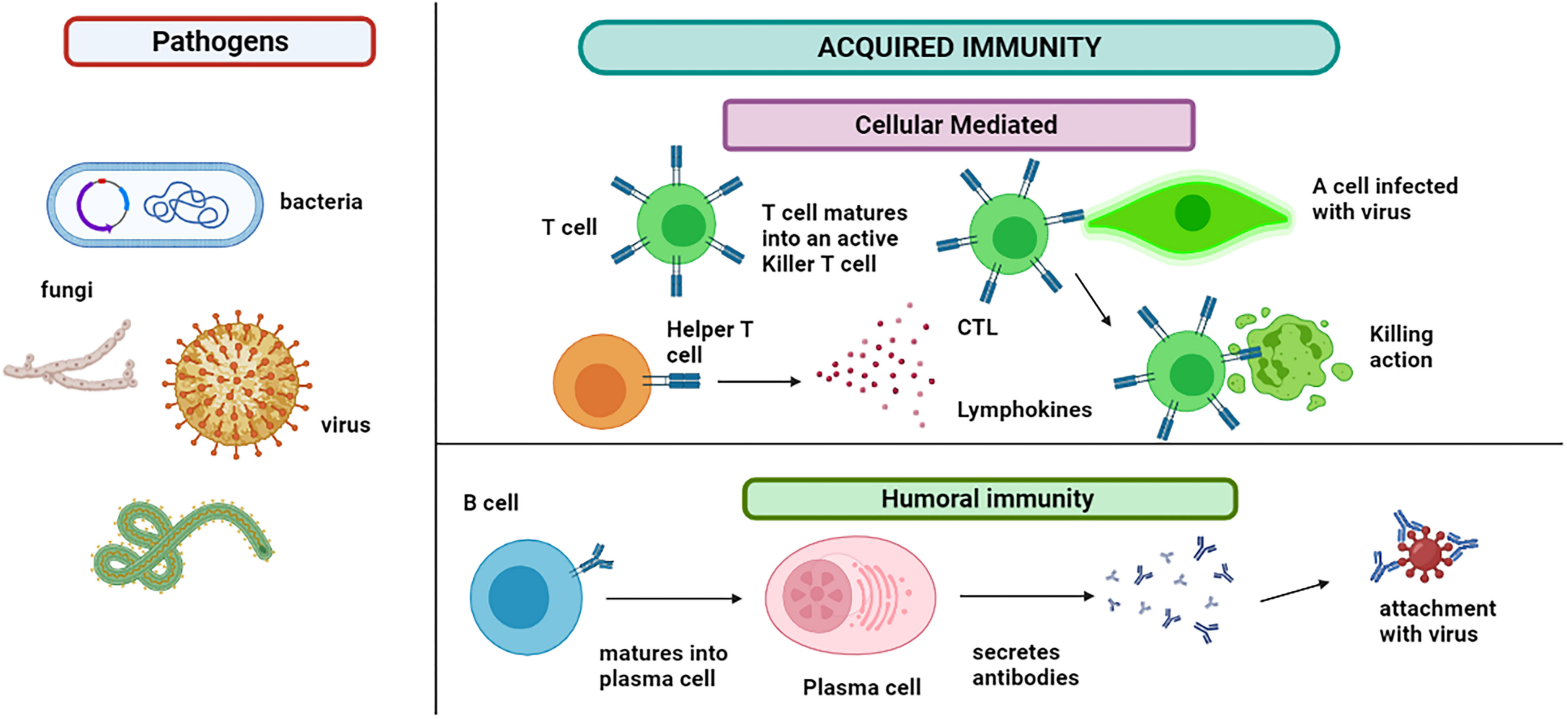
Acquired immunity: immune cells show two types of responses. Humoral immunity involves the B cells that mature into plasma cells that secrete antibodies and attach to the pathogen. B cells also produce memory cells for the future attack of the same pathogen. Cytotoxic T lymphocytes (CTLs) are immune cells that play an important role in the fight against infections and tumor immunology. T cell matures into an active killer T cell called cytotoxic T lymphocytes, which attach to the infected cell and kill it. The lymphokines attract additional immune cells for stimulation and cascade the effect.

Like PAMPs, another type of molecule, immune-activating, is recognized named damage-associated molecular patterns (DAMPs). This group of stimulators is released in the extracellular environment and activates the immune cells and sudden inflammation (64). The pathogen may or may not be killed and require more action of the immune cells (54). The acquired immunity T and B cells are responsible for modulating the antigen-specific immune response, as shown in Figure 4. These cells can recognize the antigenic epitope. The B cells are activated to plasma cells for producing the antibodies (Abs). This offshoot to humoral and cell-mediated immunity involves B cells and T cells, respectively (65).

Activating CD4 T cells by IL-4 and B cells produces an antibody response. BCR (B cell antigen receptors), a cell-bound antibody, is displayed by Naïve B cells in the lymph node site. When BCR is involved, the receptor or ligand complex is internalized by B cells. The B cells digested the antigens and then presented them as antigenic peptides upon an MHC-II receptor (66). The B cells, now called plasma cells, will consequently release the antibodies upon synapses with CD4 T cells. The antibodies are released in the blood, which binds to the pathogen and inactivates it. Few cells also contribute to forming memory cells. In case of reinfection, these memory cells react more quickly and develop immunity within 2–3 days (54).

## Beyond Dormancy and Defense

Recently, many efforts have been made to make genetically modified bacteria a therapeutic agent for treating cancer (67). Several signaling pathways are initiated to mediate the immune response and other physiological effects. IKKε is one of the signaling pathways involved in various biological processes such as innate immune signaling, activation of T cells, inflammation, tumor formation, and diseases related to metabolism. It is also involved in signal transduction involving innate and adaptive modes of immunity. IKKε is stimulated to add phosphorus group to nuclear factor of activated T cells (NFAT) proteins, which restricts T cell activation (68). Viruses target IKKε and inhibit the IKKε-mediated antiviral immunity to counteract the effect. Hence, IKKε modulators can help treat various chronic infectious diseases, inflammation, autoimmune disorders, and malignancies (69). *In vivo* studies were demonstrated when antibiotics treatments were given (70).

In addition to showing microbicide functions, Nitric oxide also plays a role in performing the immune system's actions. The actions of NO production after the viral infection are no less complex. In addition, to show the effect, NO also has harmful outcomes on the host (71, 72). Hence, complete knowledge of NO in disease pathogenesis and immunomodulation would be helpful to develop vaccines and design certain therapeutic strategies (73). It is also reported that the movement of certain ions such as Zn, Cu, and Fe are involved in controlling inflammation and innate immune function. Hence, Cu and Zn toxicity is important to clear pathogens from the body (72). Zn trafficking inside the cell increases the capability of the macrophages and neutrophils to show bactericidal effects (74). The elevated level of Cu is associated with the production of a ferroxidase named ceruloplasmin, an important part of Fe homeostasis. The Fe transport is linked with an inflammatory response (75).

## Immune Receptor Checkpoint Proteins

Certain types of signaling proteins are involved in the detection of potential infections. They are sometimes referred to as immune checkpoint proteins. It includes pattern recognition receptors (PRRs) that inquire the extracellular and the intracellular microenvironment (76) PRRs include TLR family proteins such as Dectin of C-type lectin receptors. These receptors have a nucleotide-binding domain with leucine-rich repeats such as NLRs, AIM2-like receptors (ALRs), and RIG-I-like receptors (5). Macrophage-Inducible C-Type Lectin (Mincle) and Dectin-2 do not fall under this category (77). The pathogenic components of the microorganisms are recognized by Toll-like receptors (TLRs) that act as the main defense mechanism against the infection (78). The TLRs are present on various cells, such as immune cells, macrophages, dendritic cells, natural killer cells, lymphocytes, and non-immune cells, including fibroblasts, nerve cells, and myocardiocytes (79).

TLRs are classified into two subfamilies based on their subcellular localization; plasma membrane TLRs include TLR1, TLR2, TLR4, TLR5, TLR6, and TLR10; and Endosomal TLRs include TLR3 and TLR7 to 9 (76) TLRs located on the surface of cells mainly recognize components of microbial cell membranes such as lipids, lipoproteins, and proteins. Intracellular TLRs recognize nucleic acids derived from bacteria and viruses (79). On detection of these ligands, many signaling pathways are initiated that activate AP-1 (an inflammatory transcription factor), NF-Kb family, and others. These factors initiate or express the immune response (5).

The danger signals and microbial products can also be recognized by nucleotide-binding oligomerization domain (NOD)-like receptors (NLRs) (80). The NLRs detect the ligands from the pathogen, such as lipopolysaccharides (LPS), flagellin, and nucleic acid. It also recognizes host cell and environmental sources such as skin irritants and UV-radiation. NLRs and PRRs recognize these ligands and activate certain inflammatory responses. Few NLRs can also respond to cytokines as in interferons (81). NLRs are divided into four subtypes based on the N-terminal domain configuration (79). When NLRs are activated, they exert various physiological effects such as initiating the downstream signaling for culminating the responses to inflammation, assembly of inflammasomes, NF-kB pathway activation, and transcriptional activity (80). NLRP receptors possess an association with inflammasomes. DAMP and PAMP signaling mediates the inflammatory response through a macro protein complex named inflammasomes. Firstly, procaspase-1 is activated to caspase-1, a series of inflammatory cytokines [such as-1b and IL-180 and high mobility group box one protein are activated (79)].

Another factor is AIM2 which acts as a cytoplasmic sensor of dsDNA from pathogens. It recruits ASCs. It also recruits caspase-1 that eventually forms the AIM2 inflammasomes (82). It gets activated to provoke an inflammatory response employing cytokine maturation and cell death (7). The immune responses initiated to fungal infections involve the Dectin-2 particularly the innate immune response (83).

## Reprogramming of the Immune System

The preliminary immune system of the embryo and the fetus is not uniform with an adult immune system as different cells reside in them. The immune cells are programmed during their fetal development, but they can be reprogrammed afterwards during puberty (84). Different immune cell populations emerge at different periods of life by differentiation. The chances of reprogramming (imprinting) persist during differentiation (9). The brain could also be involved in imprinting during the later developmental periods or the whole life (85). The development of asthma, allergic rhinitis, eczema, and wheezing can be enhanced by perinatal maternal anxiety during pregnancy. It also increases the effects of chemical stressors, such as traffic-related air pollution (86). Emotional stress can also worsen asthma (87). Defective reprogramming in puberty and perinatal imprinting could have similar effects, but the effect period is brief in the former, considering the stimulation time (9). Stressors can induce epigenetic reprogramming in this time, leading to autoimmune disorders (88).

Since birth, external and internal stimuli constantly challenge the immune system (51). A footprint is produced by the confrontation between stimulus and our immune cells, which modify the original naive immune system that will gradually learn to discriminate between non-toxic and decisive pathogenic stimuli (89). Immunological memory has long been thought to exist in the adaptive immune system. This system develops long-standing memory cells from B and T lymphocytes upon confronting a new antigen (51). Later studies have shown that immunological memory depends not only on lymphocytes (51). Upon encounter with stimuli, the functions of cells like monocytes, macrophages, dendritic cells (DCs), and NK cells, which are a part of the innate immune system, are also affected. This results in physiological reprogramming, providing a quicker and more effective reaction to future threats (90, 91).

The long-term reprogramming of the innate immune cells can occur by different mechanisms. It occurs mainly by innate cell metabolism or the long-epigenetic markers. Glycolysis and oxidative phosphorylation are the main focus of many studies on innate immune metabolism (51). Immune cells have a minimal biosynthetic requirement. Their metabolic pathways are oriented toward glucose metabolism *via* glycolysis and oxidative phosphorylation (92). When they are activated, glucose uptake increases, and it converts into lactate by aerobic glycolysis, forming precursor molecules needed for the synthesis of amino acids, nucleotides, and lipids (93). The tricarboxylic acid cycle (TCA) is mostly used by resting circulating monocytes. Its products are used for oxidative phosphorylation or biosynthetic products for other molecules (51). The cellular metabolism shifts from oxidative phosphorylation to aerobic glycolysis when a fungal component like glucan induces trained immunity. This process involves the protein kinase B (Akt)/mammalian target of rapamycin (mTOR)/hypoxia-inducible factor 1 (HIF1) pathway, which is required for successful induction of trained immunity (90, 94). Many epigenetic modifications are closely linked to metabolic processes. For example, metabolites like substrates or cofactors are required by histone-modifying enzymes. DNA/histone methyl transferases require S-adenosyl methionine for its right functioning (95, 96). When terminally differentiated cells, as monocytes and macrophages, are confronted with pathogens, their histone acetylation and methylation signals change. This influences their gene expression patterns upon successive stimulation (90, 94). After subsequent stimulation, LPS produces histone changes that suppress pro-inflammatory gene expression (a characteristic of immunological tolerance) (51).

In immune-mediated diseases, modulation of cellular reprogramming is thought to have great therapeutic potential (51). In the last years, studies have shown how long-term reprogramming of cells is critical for early cytokine production, inducing immune-paralysis, and creating disease tolerance after pathogen exposure (10).

## The Immune Revolution

An immune privilege is a form of biology without immune-editing (97). The Innate immune system does not discriminate between self and non-self but instead responds to danger signals. These danger signals could be either endogenous or exogenous. PAMPs refer to exogenous danger signals DAMPs refer to endogenous danger signals (98). PAMPs are distinctly retained patterns in microbial pathogens. DAMPs are Proteins, chemokines, cytokines, and compounds produced by distressed and damaged cells. PAMPs and DAMPs can activate the innate immune response with the help of PRRs present on immune-responsive cells (99). This activates many signaling cascades, which induce responses against cell damage (77). It may be particularly noticeable in oncogene causing cancers in which cancers establish immunosuppression in the initial phases of the disease (97). According to the current understanding of tumor immune-surveillance, T cell detection of cancer antigens results in tumor clearance, except when cancer undergoes immune-editing (e.g., deletion of MHC or the antigen) or immunosuppressive pathways evolve to decrease T cell reactivity (100). The use of checkpoint inhibitors in modern immunotherapy has revolutionized the patient care of adults with advanced cancer. Inhibition of Programmed cell death receptor-1 (PD-1) and Cytotoxic T lymphocytes associated protein 4 (CTLA-4) pathways have been demonstrated to improve survival in different diseases (101). The monoclonal antibody ipilimumab, which blocks CTLA-4, was the first treatment to increase overall survival in patients of metastatic melanoma, and it has received international approval for that malignancy (102). The technique of targeting the PD-1 receptor on T lymphocytes, from which tumor cells defend themselves by expressing the PD-1 ligand (PD-L1), was developed due to growing knowledge regarding the release of immune inhibitory checkpoints (102). Treatment of more than 30 different cancers by using Antibodies that block PD-1 or PD-L1 is in clinical development (11).

Frequent T-cell mediated inflammatory conditions of the skin are Psoriasis and atopic dermatitis (AD) (103). The disorders are similar in the concept that epidermal keratinocytes alter their growth and differentiation responses in response to T-cell-produced cytokines (103). Psoriasis is a skin condition characterized by a patterned response to stimulated immune cells and cytokines. The most recent disease model assumes that the Type 17 (Th17, Tc17) T-cell is the main factor of psoriasis and that certain auto-antigens are probable activators (104, 105). Tissue necrosis factor (TNF) and IL-17 (IL-17A/IL-17F), IL-26, IL-29 are the cytokines that are synthesized by type 17 T cells (106, 107). CCAAT/enhancer-binding protein (C/EBPb) or d, NFkB, and Signal Transducer and Activator Of Transcription 1 (STAT1) are activated by cytokines in keratinocytes and other skin cells. It results in wide feed-forward inflammatory reactions that self-intensifies and cause stimulation and recruitment of Th1 and Th22 T-cell subtypes to psoriatic lesions (108). The differentiation of Type 17 T-cells is directed by proteins encoded by various psoriasis inherited risk alleles. As a result, we believe psoriasis is primarily an example of polar Type 17 immunity that makes distinct tissue phenotype (109). It is supported that ninety percent of psoriasis Vulgaris patients may be effectively medicated with particular IL-17 antagonists (105). IL-17 or IL-17 receptor antibodies, developing IL-23 antagonists, TNFα antagonists, and ustekinumab (a combination IL-12/IL-23 antagonist), are now highly efficient therapies for moderate-to-severe psoriasis (110). AD is a disease caused by multiple polar immune pathways resulting in various disease characteristics. In major subtypes of AD, two T-cell types, i.e., Th2 and Th22, frequently occur and are stimulated (103). The allergic relationship with AD maybe denotes the chronic stimulation of Th2 T-cells with IgE class switching induced by IL-4 and IL-13 (111). AD can be effectively suppressed by wide inhibition of T-cell stimulation by using cyclosporine. However, due to renal toxicity, this medicine may only be administered for short periods of time. Withdrawal symptoms are also common after its discontinuation (112). With the recent validation of dupilumab, an antibody to IL-4 receptor that inhibits IL-4 and IL-13 receptor binding, significant progress has been achieved (113–115).

## Challenges in Studying Immune Tolerance and Resistance

Immune tolerance-inducing strategies are alike over various immune disorders, like allergy, autoimmunity, and allograft rejection. It is difficult to modify the immune response for therapeutic advantage while bypassing long-term immunosuppression to treat immune-mediated diseases (116). Efforts to generate immunological tolerance represent targeted and particular ways to reprogram, regulate, or selectively ablate immunological cells, eliminate hazardous responses, and restore immunological homeostasis (117). Several drugs are now available which induce immune tolerance. These drugs target T cells and include monoclonal antibodies, cell surface proteins, and soluble ligands (117). Many of these medications work by decreasing T cells (e.g., anti-CD52 monoclonal antibody, CAMPATH-1H) or reducing their function generally (e.g., calcineurin inhibitors), and thus significantly increase risks for toxicity, particularly infection (118). It's dangerous to cause such drastic changes in the immune system, and it might not be important to re-establish homeostasis (12).

PRRs can identify many PAMPs and DAMPs during an innate immune response, resulting in type I/III interferon (IFN), NF-κB signaling pathways, and inflammasomes. It leads to pro-inflammatory and antiviral reactions in innate immunity and adaptive immunity. Because persistent stimulation of inflammation and IFN might harm host cells, their activity must be properly controlled (119). Many subsequent NF-B genes and type I IFN signaling can negatively modulate these pathways through negative feedback mechanisms, regulating cell tolerance to persistent activation (120). Also, molecular positioning and density of major receptors and regulators of the innate immune system, which regulates innate immune tolerance, can be changed by other cellular activities. These cellular activities include endocytosis, autophagy, and the proteasome breakdown pathway (3).

Biomarkers of disease severity and clinical efficacy are essential because they can provide vital insights into fundamental mechanisms of the main disease, perhaps leading to new treatment techniques (121). However, most of those biomarkers investigated concerning immune-mediated disorders so far have shown low predictive accuracy (122). For instance, Serum creatinine, in kidney transplantation, could be used as a biomarker for the immunological response to the transplant. It offers minimal to zero predictive precision for a patient's future status and provides little insight into pathways of rejection or tolerance (12).

Successful allergen-specific immunotherapy (AIT) results in long-term tolerance to allergens. It leads to a reduction in symptoms requirement for pharmacotherapy, finally making life better for patients with immunoglobulin IgE-mediated illnesses (123). Even though AIT's disease-modifying effect and high efficiency have changed the therapy of allergic disorders, some people are insensitive to the treatment (43). Local nasal immunotherapy (LNIT) appears beneficial only for rhinitis symptoms and needs a specific dosing strategy (43). The use of LNIT is diminishing as a result of these technological challenges. The IDIT is another method of administering AIT that involves injecting allergens into the skin's dermis. It is both safe and effective after just a few doses; nevertheless, it has been observed to aggravate respiratory allergy symptoms as a side effect (124). However, various limitations have been identified, which could significantly impact the conclusion of such a comparison. These limitations include patient heterogeneity, geographic variation in allergen exposure, and research standards among earlier and recent studies (125). Though tremendous progress has been made in apprehending the mechanisms of AIT, the cellular and molecular alterations that underlie the formation of allergen tolerance remain a mystery (126).

Immunotherapy has resulted in clinical advantages in the treatment of some cancer patients. However, the primary issue in cancer treatment is disease progression and drug resistance after medication (127). One type of resistance is Darwinian natural selection which results from preexistent genetic or epigenetic features selected in tumor mass before therapy intervention (128). The DNA and subsequent instability of the mutated cell appear to be the primary drivers of the immune-resistant cancer cells mutation generated by this mechanism. The second type of resistance is developed at the individual cancer cell level (129). It is due to the ability of tumor cells to change their gene expression in response to immune cells or their metabolites. This type of acquired resistance might rely on adaptive mechanisms and immunological homeostasis, also known as homeostatic resistance. The development of PD-L1 due to IFN-secretion in induced tumor cells is a clear example of this resistance (127). The anti-PD-L1 treatment greatly improved; nonetheless, the immune therapy encountered numerous issues (130, 131), including traditional treatments that resulted in therapeutic resistance and immune-related side effects. The most difficult challenge for immunotherapy is rationalization, but the extension of its utility is also a challenge (127).

Adaptive immune resistance refers to a process in which tumor antigen-specific T cells seek to target cancer. Still, cancer responds by changing reactively to protect itself from this immunological atta. Drew Pardoll first coined the term to describe how T cells' production of interferons in response to their cognate antigen results in responsive expression of the PD-1 ligand (PD-L1) by cancer cells and the cutting off of PD-1–positive T cells (132). This theory explains how the immune system cannot recognize tumors that are ordinarily immunogenic cancers while still defending the body from infections (133). The explanatory impact of differential PD-1 and PD-L1 expression has yet to be fully understood, while some research shows that high expression is associated with improved immunotherapy success. As immunotherapy advances, a better knowledge of drug resistance mechanisms will be required, facilitating the development of new strategies to overcome significant and acquired anti-PD/PDL-1 antibody resistance (127).

## Perspectives

Arithmetic modeling can give a rational framework for underpinning and combining different cross-disciplinary undertakings. This is particularly important in AMR research, where various elements link in complex methods. Arithmetic modeling, for example, can be used to investigate the underpinning mechanisms that drive fundamental microbiology along with patterns in the occurrence of drug resistance infections (134). Many other scientific strategies, such as the synthesis and revelation of new antimicrobials, can also help to reduce AMR (135).

Interference between NK and DCs could be a key factor in the immune system's overall response to malignancies and viruses (136, 137). Evidence suggests that activated NK cells regulate cytokine-producing Th cells, Th cell polarity, DC motility, and stimulatory actions (138). On the other hand, the effector actions of NK cells may depend on excitatory relationships with mature DCs (138). According to a study, L19mTNF therapy with melphalan decreased Treg cells and triggered a long-standing T-cell-mediated immunological response (139). This NK-DC-Th-Tc process may also be active when L19mTNF is combined with IL-2 or gemcitabine and may provide pointers for susceptible immune resistant cancers to checkpoint blockade immunotherapy (140). It's noticed that the pairing of FOLFOX with bevacizumab can diminish granulocyte MDSCs, expand in recurrence of inflammatory helper T cells (Th17), and yield a favorable milieu for immune checkpoint inhibitor medication (141). The impact of chemotherapy and anti-angiogenic agents on immune checkpoint treatment is being studied comprehensively.

The potential effect of immunotherapy on resilient clone cells and the shortage of intended genome editing are being thoroughly investigated (142). There is a shortage of information about the particular steps leading to tumor formation, malignancy, and resistance. Several processes supply metabolic routes; however, it is unclear whether they are also involved in upstream signaling pathways (143). As a result, future research should determine the T cells' regulatory response based on the relevant immunological pathways. Furthermore, future work should recognize particular cancer antigens using a highly customized technique and devise an effective lymph-depleting strategy before T-cell moves (127).

## Conclusion

The immune response status correlates with the time of onset of host-pathogen interaction, variants and exposure to the host attackers, the duration and the initiated response of the body, and the underlying mechanisms in developing resistance to certain invaders, which otherwise would prove to be a big threat for life expectancy and survival is of utmost importance. The modulation of cellular programming is believed to have great potential as a therapeutic tool and novel clinical approaches. The convention of arithmetic modeling would prove to be a revolutionary tool in AMR research as it showcases the host's underpinning cellular mechanisms and drug resistance patterns. There is still a need to develop research methodologies to identify the peculiar behavior of the regulatory T cells to identify relevant immunological pathways, which would be a turning point for tumor formation, malignancies, and disease resistance in the future.

## Data Availability Statement

The raw data supporting the conclusions of this article will be made available by the authors, without undue reservation.

## Author Contributions

HA, JC, and NA design the study. AJ, ZJ, MH, JB, ZA, and IN wrote the article. ZA, NM, AJ, HA, and JC designed the figures. AJ, JC, ZA, and HA edited the article. All authors contributed to the article and approved the submitted version.

## Conflict of Interest

The authors declare that the research was conducted in the absence of any commercial or financial relationships that could be construed as a potential conflict of interest.

## Publisher's Note

All claims expressed in this article are solely those of the authors and do not necessarily represent those of their affiliated organizations, or those of the publisher, the editors and the reviewers. Any product that may be evaluated in this article, or claim that may be made by its manufacturer, is not guaranteed or endorsed by the publisher.

## Abbreviations

DCs: dendritic cells
NK: natural killer cells
NLRs: NOD-like receptors
AIM2: absent in melanoma two protein
DAMP: damage-associated molecular pattern
HSP: heat-shock protein
PD-1: programmed cell death receptor-1
IPAF: ICE protease-activating factor
IRAK4: interleukin-1 receptor associated kinase 4
KIR: killer-cell immunoglobulin-like receptors
TNF: tissue necrosis factor
LBP: LPS binding protein
LPS: lipopolysaccharide
LRR: leucine-rich repeat
AIT: allergen-specific immunotherapy
MAL: MyD88 adapter-like
MBL: Mannose-binding lectin
NLR: nucleotide oligomerization domain (NOD)-like receptors
PAMP: pathogen-associated molecular pattern
PRR: pattern recognition receptor
RAGE: receptor of advance glycation end product
SNP: single nucleotide polymorphism
TIR: toll/interleukin-1 receptor-like domain
TIRAP: toll-interleukin 1 receptor (TIR) domain-containing adaptor protein
TLR: toll-like receptor
LNIT: local nasal immunotherapy
AMR: antimicrobial resistance
MDSCs: myeloid-derived suppressor cells.
